# Biochemical and molecular characterization of sialylated cervical mucins in sheep^†^

**DOI:** 10.1093/biolre/ioac077

**Published:** 2022-04-26

**Authors:** Laura Abril-Parreño, Jack Morgan, Anette Krogenæs, Xavier Druart, Paul Cormican, Mary E Gallagher, Colm Reid, Kieran Meade, Radka Saldova, Sean Fair

**Affiliations:** Laboratory of Animal Reproduction, Department of Biological Sciences, School of Natural Sciences, Biomaterials Research Cluster, Bernal Institute, Faculty of Science and Engineering, University of Limerick, Limerick, V94 T9PX, Ireland; Animal & Bioscience Research Department, Teagasc, Animal & Grassland Research and Innovation Centre, Grange, C15 PW93, Ireland; NIBRT GlycoScience Group, The National Institute for Bioprocessing Research and Training, Blackrock, Dublin, A94 X099, Ireland; Faculty of Veterinary Medicine, Department of Production Animal Clinical Sciences, Norwegian University of Life Sciences, Ås 5003 1432, Norway; UMR-PRC, INRA-85, Université de Tours, IFCE, Department of Physiologie de la Reproduction et des Comportements, Institut National de la Recherche Agronomique, Nouzilly 37380, France; Animal & Bioscience Research Department, Teagasc, Animal & Grassland Research and Innovation Centre, Grange, C15 PW93, Ireland; UCD Veterinary Sciences Centre, University College Dublin, Belfield, Dublin 4, D04 W6F6, Ireland; UCD Veterinary Sciences Centre, University College Dublin, Belfield, Dublin 4, D04 W6F6, Ireland; UCD School of Agriculture and Food Science, University College Dublin, Belfield, Dublin 4, D04 W6F6, Ireland; NIBRT GlycoScience Group, The National Institute for Bioprocessing Research and Training, Blackrock, Dublin, A94 X099, Ireland; UCD School of Medicine, College of Health and Agricultural Science, University College Dublin, Belfield, Dublin 4, D07 A8NN, Ireland; CÚRAM, SFI Research Centre for Medical Devices, National University of Ireland, Galway, H91 W2TY, Ireland; Laboratory of Animal Reproduction, Department of Biological Sciences, School of Natural Sciences, Biomaterials Research Cluster, Bernal Institute, Faculty of Science and Engineering, University of Limerick, Limerick, V94 T9PX, Ireland

**Keywords:** sialic acid, cervical mucus, sperm transport, ewe breed, estrus cycle, fertility

## Abstract

Sialic acid occupies terminal positions on *O*-glycans of cervical mucins, where they contribute to the increased viscosity of mucin thereby regulating sperm transport. This study characterized the sialylated cervical mucins from follicular phase mucus of six European ewe breeds with known differences in pregnancy rates following cervical artificial insemination (AI) using frozen–thawed semen at both synchronized and natural estrus cycles. These were Suffolk (low fertility) and Belclare (medium fertility) in Ireland, Ile de France and Romanov (both with medium fertility) in France, and Norwegian White Sheep (NWS) and Fur (both with high fertility) in Norway. Expression of mucin and sialic acid related genes was quantified using RNA-sequencing in cervical tissue from Suffolk, Belclare, Fur, and NWS only. Cervical tissue was also assessed for the percentage of cervical epithelial populated by mucin secreting goblet cells in the same four ewe breeds. Biochemical analysis showed that there was an effect of ewe breed on sialic acid species, which was represented by Suffolk having higher levels of Neu5,9Ac_2_ compared with NWS (*P* < 0.05). Suffolk ewes had a lower percentage of goblet cells than Fur and NWS (*P* < 0.05). Gene expression analysis identified higher expression of *MUC5AC*, *MUC5B*, *ST6GAL1*, and *ST6GAL2* and lower expression of *ST3GAL3, ST3GAL4*, and *SIGLEC10* in Suffolk compared with high fertility ewe breeds (*P* < 0.05). Our results indicate that specific alterations in sialylated mucin composition may be related to impaired cervical sperm transport.

## Teaser

Poor cervical sperm transport in some ewe breeds following cervical artificial insemination using frozen–thawed semen could be due to differences in cervical sialic acid composition and the proportion of neutral and sialylated *O*-glycans.

## Graphical Abstract

**Graphical Abstract:**
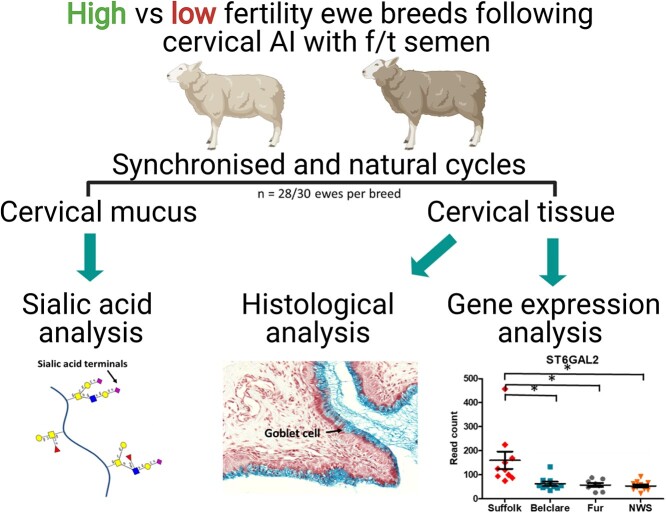

## Introduction

Cervical mucus is a complex viscoelastic gel secreted by the cervical epithelium where it acts as a medium for lubrication and it maintains a cervical mucosal barrier against ascending pathogens from the vagina while also allowing sperm migration in the lead up to ovulation [1]. Cervical mucus is mainly composed of water (95% by weight) and gel-forming mucins, which are large glycoproteins that represent >80% of the mucus organic fraction and are encoded by mucin genes [2]. In the bovine cervix, two gel-forming mucins (*MUC5AC* and *MUC5B*) and four membrane-bound mucins (*MUC1, MUC16, MUC20*, and *MUC4*) have been identified [3], whereas two more gel-forming mucins (*MUC2* and *MUC6*) have been reported to be expressed in the human cervix [4]. A number of studies have investigated how mucin gene expression changes across the menstrual [5] and estrus [3] cycle, showing a peak around the time of ovulation. Mucin core proteins are modified by the action of glycosyltransferases, which add sugar residues to the hydroxyl groups of threonine and serine [6]. Most of the *O*-glycans have terminal additions that carry fucose (Fuc) and sialic acid (*N*-acetylneuraminic sialic acid (Neu5Ac) and *N*-glycolylneuraminic sialic acid (Neu5Gc)) terminals.

Sialic acid terminals aid in the protection of the cervix from bacterial glycosidases and proteases [7]. In addition, mucus rheology and hydration seems to be related with the presence of negatively charged terminal groups as the sialic acids on *O*-glycans [8]. Andersch-Björkman et al. [5] reported that cervical mucus around the time of ovulation contain more neutral and less acidic mucins, resulting in a more hydrated and less viscous mucus, which facilitates sperm penetration around the time of ovulation. Sialic acid also facilitates tolerance by female innate pattern-recognition molecules, masking potential antigenic sperm molecules [9]. Apart from masking receptors, it has been demonstrated that bovine sialylated cervical mucins have an anti-inflammatory capability to modulate neutrophil extracellular traps (NETs), which are released by neutrophils in order to combat invading pathogens, although NETs can also damage endogenous cells [10].

In sheep, cervical artificial insemination (AI) with frozen–thawed semen yields unacceptably low (< 30%) pregnancy rates worldwide [11, 12]. The exception is in Norway where vaginal (shot-in-the-dark) AI of Norwegian White Sheep (NWS) yields non-return rates in excess of 60%, which have been consistently reported [13, 14]. This is likely due to the breed of the ewe used in Norway as Donovan et al. [15] reported significant ewe breed effects in fertility with pregnancy rates of 18, 28, 44, and 77% for Suffolk, Texel, Belclare, and Finnish Landrace ewes, respectively, following cervical AI using frozen–thawed semen. In addition, this study compared the procedures used in Norway under Irish conditions and reported that pregnancy rates did not differ between ewes inseminated with Irish frozen–thawed semen and ewes inseminated with Norwegian frozen–thawed ram semen. The primary reason for the differences in pregnancy rates between ewe breeds has been shown to be due to the inability of frozen–thawed ram sperm to traverse the cervix in some ewe breeds such as the Suffolk, but not others [16].

Richardson et al. [17] reported that glycosylation of cervical mucins differs between Suffolk (low fertility) and Belclare (medium fertility), with higher sialic acid content in the cervical channels (where sperm progression occurs) of Suffolk compared with Belclare. In addition, there was higher expression of the sialyltransferase *ST6GAL1* in the cervical channels of Suffolk compared with Belclare ewes, which supports the evidence of the role of the sialic acid in the inhibition of sperm transit through the cervix. Indeed when exogenous sialic acid was added to mucus in vitro ram sperm penetrate was inhibited due to sialic acid-binding sites on sperm being taken up by the free sialic acid [17].

In this study, we aimed to characterize the sialic acid species and the proportion of neutral and acidic *O*-glycans of the cervical mucins at the follicular phase of both synchronized and natural estrus cycle in ewe breeds with known differences in pregnancy rates following cervical AI using frozen–thawed semen. In addition, we assessed the gene expression of key genes involved in mucin biosynthesis and sialylation of the cervical tissue in a subset of the ewe breeds. Cervical tissue samples were also used to assess the percentage of goblet cells along the cervical epithelium.

## Material and methods

### Ethical approval

Protocols were developed in accordance with the *Cruelty to Animals Act* (Ireland 1876, as amended by European Communities regulations 2002 and 2005) and the European Community Directive 86/609/EC. In Ireland, the study was approved by the Teagasc Animal Ethics Committee (TAEC145/2017) and all animal procedures performed were conducted under experimental license from the Health Products Regulatory Authority (AE19132/P065). In Norway, the study was approved by Norwegian Food Safety Authority (FOTS ID 13168). In France, the study was approved by the ethics committee and the Ministry of Research.

### Experimental design

The aim of this experiment was to characterize the sialic acid species of the cervical mucins in six ewe breeds with known differences in pregnancy rates following cervical AI using frozen–thawed semen. This study was part of larger project that aimed to interrogate the ewe breed differences in cervical mucus properties and anatomical characteristics across the estrus cycle of both synchronized (using progestogen pessaries combined with equine chorionic gonadotropin) and natural cycles. A description of the experimental model, animal treatments, and sample collection has been previously reported by Abril-Parreño et al. [18].

**Figure 1:**
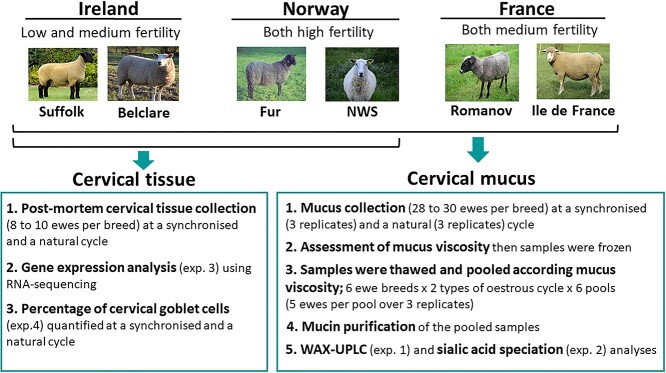
Experimental flow diagram of cervical tissue and mucus collection, sample preparation and analysis. NWS = Norwegian White Sheep. Exp. = experiment.

Briefly, cervical mucus samples were collected from six ewe breeds with known differences in pregnancy rates following cervical AI using frozen–thawed semen. These were Suffolk (low fertility) and Belclare (medium fertility) in Ireland, Ile de France and Romanov (both with medium fertility) in France, and NWS and Fur (both with high fertility) in Norway. At the natural cycle, all ewes were checked twice daily for signs of estrus over a 6-day period using a teaser ram with an apron fitted (no semen/seminal plasma was allowed to be deposited into the vagina of the ewe). At the synchronized cycle, ewes were synchronized using intravaginal progestagen vaginal sponges (20-mg Flugestone Acetate; Chronogest vaginal sponges, Intervet, Boxmeer, The Netherlands), inserted on a random day of the cycle. After 14 days, the sponges were removed and ewes were treated with equine chorionic gonadotropin (400 IU; Intervet, Boxmeer, The Netherlands). We used this established protocol that works in >95% of ewes (during the breeding season) based on previous studies [19, 20]. Synchronized ewes were not heat checked as is the norm for most inseminated in fixed AI programmes, and cervical mucus was collected at a time (56 h) when ewes would normally be cervically artificially inseminated in time-fixed AI programmes using frozen–thawed semen. Cervical mucus was collected from each ewe at the follicular phase of both a synchronized (56-h post pessary removal) and a natural estrus cycle (12-h post detection of standing estrus), which was then replicated three times with the same ewes over a period of approximately 6 months (Figure 1). After each mucus collection, samples were transported to the laboratory (maximum time = 1 h) for assessment of cervical mucus properties (weight, viscosity, and color) following which the samples were stored at −20°C. Values of mucus weight, viscosity, and color as well as a more detailed description of the experimental model can be found at Abril-Parreño et al. [18]. Mucus samples were ranked according to viscosity at collection as assessed by the time in seconds for 4 μL of mucus to fill a Leja 20-μm deep chamber slide (IMV Technologies, L’Aigle, France). The more viscous mucus was, the longer it took to fill the chamber. A maximum of 420 s was allowed per sample for mucus to fill the chamber. Following thawing, samples were pooled according to viscosity post collection to ensure enough mucin for analysis. For the synchronized animals, each pool consisted of mucus from five animals (five ewes where their average viscosity was highest pooled together, then the next five and so on) of the same breed collected over three replicates (Supplemental file 1). Each pool was a total of 15 samples consisting of five ewes, each with one sample collected at each of three replicates/cycle. We then pooled mucus (1 pool = 15 samples) from the same five animals for the natural cycle using the same mucus viscosity ranking as the synchronized cycle. The three replicates were three separate cycles at both a natural and a synchronized cycle with one mucus collection at the follicular phase of each cycle. In all, the sample collections took ~6 months to complete (3 synchronized cycles and 3 natural cycles = 6 cycles). Mucins were purified from cervical mucus samples using the described method in Supplemental file 2. Following slaughter to collect cervical tissue (experiment 3 and 4), the ovaries were assessed for the presence or absence of dominant follicles (without an active corpus luteum) and/or fresh ovulations as evident by a corpus hemorrhagicum. These structures were present in all the ewes of this study confirming they responded successfully to the sponge. For experiments 3 and 4, cervical tissue samples from two Irish ewe breeds (Suffolk and Beclare) and two Norwegian ewe breeds (NWS and Fur) at the follicular phase of both synchronized and natural estrus cycle were collected and later subjected to RNA-sequencing analysis. For this study, specific mucin and sialic acid related genes were interrogated from the data set generated in our RNA-sequencing analysis. To assess the percentage of the epithelium consisting of goblet cells we used cervical tissue sections from Suffolk, Belclare, Fur, and NWS at the follicular phase of both a synchronized and a natural estrus cycle.

### Experiment 1: Weak Anion Exchange Chromatography—Ultra-Performance Liquid Chromatography Analysis

The objective of this experiment was to profile the abundance of neutral and acidic *O*-glycans in one pooled sample for each breed and type of estrus cycle. One pooled sample for each of the six ewe breeds within each type of cycle (natural or synchronized) was used. Belclare breed at the synchronized cycle was not analyzed as there was insufficient amount of mucin remaining. *O*-glycans were released from purified mucins as described by Abril-Parreño et al. [21]. The purified mucin from cervical mucus was also used for the *O*-glycan analysis, which included ultra-performance liquid chromatography (UPLC), mass spectrometry, and exoglycosidases digestion panels [21]. 2AB labelled *O*-glycans were separated by AEC-UPLC using a Waters DEAE anion exchange, 75 × 7.5 mm i.d., 10-μm column on an Aquity H-Class HILIC-UPLC system (Waters Corporation, Milford, MA) coupled with a fluorescence detector (Waters Corporation, Milford, MA), which was set with excitation and emission wavelengths of 330 and 420 nm, respectively. Gradient separations were performed using a solvent A of 20% v/v acetonitrile (Sigma Aldrich, Arklow, Co Wicklow, Ireland) and solvent B of 0.1M ammonium acetate buffer pH 7.0 in 20% v/v acetonitrile. Samples were resuspended in 50 μL of water and an injection volume of 20 μL was used in a flow rate of 750 μL per min over a 30 min run using the following gradient: 0–5 min—100% A, 5–20 min—100% → 0% A, 20–22.5 min—0% A, 22.5–23 min—0% → 100% A, 23–30 min—100% A. A fetuin *O*-glycan standard was used for calibration.

### Experiment 2: sialic acid speciation analysis

The objective of this experiment was to assess the sialic acid species of cervical mucins from a total of six mucus pools for each ewe breed at both types of cycle, with a total of 72 samples (six mucus pools from the 6 ewe breeds at both a natural and synchronized estrus) in duplicate that generated data from 144 samples. Sialic acids were released from the purified cervical mucin samples (500 mg) using mild acid hydrolysis by incubation for 2 h at 80°C with 25 μL of 2M acetic acid. From each sample 5 μL were used for labelling of sialic acid using a LudgerTag DMB (1,2-diamino-4,5-methylenedioxybenzene) Sialic Acid Labelling kit (Ludger Limited, Culham Science Centre, Abingdon, UK). Samples were labelled with 20 μL of DMB reaction mixture, which were previously prepared using 0.7 mg of DMB and 0.4 mg sodium dithionite in mercaptoethanol (1.2M) for 3 h at 50°C. The labelling reactions were quenched by adding 475 μL of milliQ water to the samples, which were immediately frozen at −20°C. The sialic acid reference panel and the standards supplied with the Ludger Kit were also labelled with 20 μL of DMB reaction mixture.

Analysis was performed within 72 h after sample preparation to avoid DMB degradation (which occurs over time). The DMB labelled samples were separated by reverse phase (RP)-UPLC analysis using a LudgerSep-uR2, 2.1 × 50 mm, 1.9-μm column on an Aquity H-Class HILIC-UPLC system (Waters Corporation, Milford, MA) coupled with a fluorescence detector (Waters Corporation, Milford, MA). Gradient separations were performed using a solvent A of 9:7:84 acetonitrile:methanol:Milli-Q water and solvent B of ACN. An injection volume of 5 μL was used in a flow rate of 250 μL/min over a 15 min run using the following gradient: 0–7 min—100% A, 7–7.5 min—100% → 10% A, 7.5–8 min → 10% A (flow rate 250 μL/min), 8–8.5 min—10% → 100% A (flow rate 250 μL/min), 8.5–15 min—100% A (flow rate 250 μL/min). The separation temperature was set at 30°C, whereas the sample temperature was 10°C. Excitation and emission wavelengths for fluorescence detection were λ = 373 nm and λ = 448 nm, respectively. Sialic acid reference panel provided by the Ludger kit was performed creating a chromatogram with seven peaks (peak 1 = 5-*N*-glycolyl (Neu5Gc); peak 2 = 5-*N*-acetyl (Neu5Ac); peak 3 = 5-*N*-acetyl-7-*O*-acetylneuraminic (Neu5,7Ac_2_); peak 4 = 5-*N*-glycolyl-9-*O*-acetylneuraminic acid (Neu5Gc,9Ac); peak 5 = Neu5,8Ac_2_ 5-*N*-acetyl-8-*O*-acetylneuraminic; peak 6 = 5-*N*-acetyl-9-*O*-acetylneuraminic (Neu5,9Ac_2_);and peak 7 = Neu5,x,xAc_3_), which were used to quantify the relative levels of the different species of sialic acid in each sample.

### Experiment 3: gene expression analysis using RNA-sequencing

The objective of this experiment was to examine functional groups of genes related to mucin production and the expression of enzymes involved in sialic acid production and modification from an RNA-sequencing dataset describing the overall cervical gene expression analysis. Total RNA was extracted from frozen cervical biopsies (*n* = 38 at the natural cycle and 39 and the synchronized cycle) immersed in TRIzol reagent, which was homogenized using a homogenizer (Bio-gen Pro200 Homogenizer, Pro Scientific) in order to lyse the tissue. The RNA extraction was completed using the RNeasy Kit (Quiagen Ltd., Crawley, West Sussex, UK) according to the manufacturer’s instructions. Total RNA concentration was quantified using the Nanodrop ND-1000 UV–Vis Spectophotometer (NanoDrop Technologies Inc., Wilmington, DE). Quality of RNA was ascertained with the use of 2100 Agilent Bioanalyzer (Agilent Technologies, Santa Clara, CA). RNA integrity number was >7 in all samples and RNA aliquots were frozen after extraction. RNA libraries were prepared for all cervical tissue samples (Illumina TruSeq Stranded mRNA Library preparation). All libraries were sequenced on an Illumina NovaSeq sequencer. Sequencing was performed for each sample at 2 × 150 bp paired end reads (50 M reads) as previously described [22].

Raw sequence data were assessed for quality using the software FastQC (v 0.11.8) http://www.bioinformatics.babraham.ac.uk/projects/fastqc/). Data were quality and adapter trimmed using the BBDuk java package to trim Illumina adapter sequences and any low quality bases (Phred score < 20) from the 3′ end of sequence read pairs. Reads were aligned to the ovine genome Oar_v3.1 using the spliced transcripts alignment to a reference (STAR) aligner. A maximum of two mismatches with the reference genome were allowed and only uniquely mapped read pairs were retained for downstream analysis. Read counts overlapping all protein coding genes in the Oar_v3.1 Ensembl (v.95) annotations were estimated using featureCounts. To filter out lowly expressed genes, genes with less than one count per million in at least ten samples were discarded from the analysis. Remaining gene counts were normalized uses the median of ratios method as implemented in DeSeq2 (version 1.130.0) [23] to account for varying sequencing depth between samples.

### Experiment 4: histological analysis of cervical goblet cells

The objective of this experiment was to assess the percentage of cervical goblet epithelial cells in the cervical tissue of four breeds with known differences in pregnancy rates following cervical AI with frozen–thawed semen. Additional formalin fixed cervical biopsies (randomly chosen) were paraffin-embedded, sectioned at 5-μm thickness, mounted on glass slides and stained with periodic acid Schiff (PAS) and Alcian blue (AB, pH 2.5) to assess the presence of goblet cells in the cervical epithelium by an experienced histology expert according to the method used by Pluta et al. [24]. Previous stain with PAS, the sections were deparaffinised and oxidized in 1% periodic acid for 10 min, followed by several rinses in distilled water. Staining was carried out in Schiff reagent at 4°C for 15 min followed by rinsing in distilled water. Sections were then counter stained with hematoxylin for 1 min, washed, dehydrated, and mounted. Sections stained with AB pH 2.5 were previously deparaffinised and stained for 5 min with 1% AB in 3% acetic acid pH 2.5, followed by rinsing several times in distilled water. After counterstaining with 0.5% aqueous neutral red for 1 min, sections were washed, dehydrated and then mounted. We used 3–5 animals per breed. The percentage of goblet cells (cells stained in blue) was determined by counting the number of these cells in a delimited area (10 replicates per slide). Slides from both stains were analyzed using Image-Pro Premium 9.2 by Media Cybernetics (Marlow, Buckinghamshire, UK).

### Statistical analysis

#### WAX analysis

The weak Anion exchange chromatography (WAX)-UPLC profiles were separated into four fractions (S0: neutral fraction, containing non sialylated *O*-glycans, and charged fractions: S1: containing mono-sialylated, S2: di-sialylated, and S3: tri-sialylated *O*-glycans). This method separates glycans based on charge, most of charged glycans are sialic acids, but some minor glycans are also included in these charged fractions such as sulphated glycans [25]. The area under each fraction peak was expressed as a relative percentage area derived from the UPLC total profile. These data were not statistically analyzed as there was only one pooled sample per group.

#### Sialic speciation analysis

The UPLC profiles were separated into seven peaks and the area under each peak was expressed as a relative percentage area derived from the UPLC total profile. Thus, a logit transform was used to map the data onto a more Gaussian distribution as previously described by Saldova et al. [25]. Peak data were checked for normality of distribution using a Kolmogorov–Smirnov test. Multivariate analysis of variance (MANOVA) with post-hoc analysis using Tukey Honest Significant difference test was then performed using SPSS software (IBM Corp., Armonk, NY). The pooled sample of mucus was the experimental unit and the model included fixed effects for ewe breed, type of cycle (natural and synchronized), and their interaction. The comparisons were considered statistically significant when *P* < 0.05.

#### Gene expression analysis

Transcript counts were modelled by fitting the data to a negative binomial distribution using genewise dispersion estimates and differentially expressed genes were identified with a generalized linear model likelihood ratio test. Statistical tests were corrected for multiple testing using the Benjamini–Hochberg method. Differentially expressed genes (DEGs) with an adjusted *P* < 0.05 and a log_2_ fold change (FC) threshold of 1.5 were used for further differentially expressed gene data exploration.

#### Histology analysis

Data were analyzed using appropriate procedures of SPSS software (IBM Corp., Armonk, NY). Multivariate analysis of variance with post-hoc analysis using Tukey Honest Significant difference test was then performed to identify significant differences across groups. Each individual tissue sample was the experimental unit and the model included fixed effects for ewe breed, type of cycle (natural vs synchronized), and their interaction. The comparisons were considered statistically significant when *P* < 0.05. Values given are the mean ± SEM.

## Results

### Experiment 1: WAX analysis; high fertility ewe breeds have higher concentration of nonsialylated glycans in cervical mucins

The majority (75%) of the structures was in the neutral fraction (S0 fraction), containing nonsialylated glycans across the six ewe breeds at both the natural and synchronized cycle. In our previous study published [25], the overall *O-*glycan analysis demonstrated that ~60% of the total glycans were neutral structures, which supports the overall trends of the present experiment. At the synchronized cycle, NWS (high fertility) had the highest percentage of nonsialylated glycans, whereas at the natural estrus cycle Fur (high fertility) had the highest. However, these were followed by the low fertility, Suffolk, which also had a high percentage of nonsialylated structures at the synchronized cycle. Levels of charged fractions, containing mono-, di-, and tri-sialylated *O*-glycans are also shown in Table 1.

**Table 1:** Relative percentage area (%) derived from the UPLC total profile of nonsialylated (Fraction S0), mono-sialylated (Fraction S1), di-sialylated (Fraction S2), and tri-sialylated (Fraction S3) *O*-glycans in cervical mucin from six ewe breeds (Suffolk, Belclare, Ile de France, Romanov, Fur, and Norwegian White Sheep (NWS)) at the follicular phase of both a natural and a synchronized estrus cycle. One pooled samples per breed and type of cycle was analyzed by weak Anion exchange chromatography (WAX)—ultra-performance liquid chromatography (UPLC) analysis

Ewe breed	Type of cycle	Fraction S0 (%)	Fraction S1 (%)	Fraction S2 (%)	Fraction S3 (%)
Suffolk	Natural	69.9	23.9	5.1	1.2
Suffolk	Synchronized	79.6	17.0	2.6	0.7
Belclare	Natural	76.6	18.9	3.4	1.1
Ile de France	Natural	71.5	23.2	4.3	1.0
Ile de France	Synchronized	71.5	22.9	4.6	1.0
Romanov	Natural	64.7	28.1	6.2	0.9
Romanov	Synchronized	79.4	17.5	2.5	0.6
Fur	Natural	86.5	11.2	1.8	0.4
Fur	Synchronized	72.2	23.1	3.9	0.7
NWS	Natural	82.6	13.2	2.3	1.8
NWS	Synchronized	91.4	7.0	1.2	0.4

^*^Belclare breed at the synchronized cycle was not analyzed as there was insufficient amount of mucin remaining to perform this analysis. NWS = Norwegian White Sheep.

### Experiment 2: sialic acid speciation analysis identified the highest levels of Neu5,9Ac_2_ in the low fertility Suffolk breed

Relative quantification of the detected sialic acids demonstrated that there were two major peaks corresponding to 5-*N*-glycolyl- (Neu5Gc) and 5-*N*-acetyl- (Neu5Ac); which were glycolylated and acetylated, respectively. These two major peaks represented 90–94% of the total sialic acid content of each sample. Minor peaks corresponding to 5-*N*-acetyl-7-*O*-acetylneuraminic (Neu5,7Ac_2_); 5-*N*-glycolyl-9-*O*-acetylneuraminic acid (Neu5Gc,9Ac); 5-*N*-acetyl-8-*O*-acetylneuraminic Neu5,8Ac_2_; 5-*N*-acetyl-9-*O*-acetylneuraminic (Neu5,9Ac_2_); and Neu5,x,xAc_3_ were also detected (Supplemental Figure S1).

**Figure 2:**
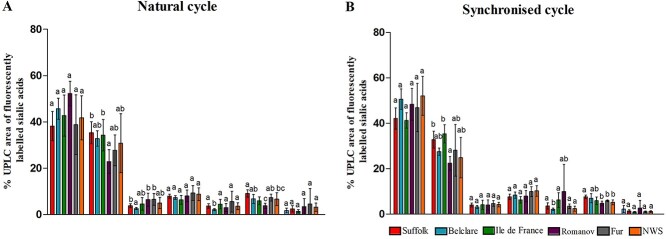
Relative percentage area derived from the UPLC total profile of seven sialic acid species (Neu5Gc, Neu5Ac, Neu5,7Ac_2_, Neu5Gc,9Ac, Neu5,8Ac_2_, Neu5,9Ac_2_, and Neu5,x,xAc3) in cervical mucins at the follicular phase of a synchronized (A) and a natural estrus cycle (B) from Suffolk (low fertility) Belclare, Ile de France, Romanov (all medium fertility), Fur and Norwegian White Sheep (NWS; both high fertility) ewes as assessed using RP–ultra-performance liquid chromatography (UPLC). Peak data were analyzed using a multivariate analysis of variance (MANOVA) with post-hoc analysis using Tukey’s Honest Significant difference test. Values are mean ± SEM. ^abc^ Different superscripts differ significantly within sialic acid species (*P* < 0.05).

There was no interaction between ewe breed and type of cycle (*P* > 0.05) for any of the detected sialic acids. Despite Neu5x,xAc_3_ being the least prevalent sialic acid detected there was a higher content at the natural cycle compared with the synchronized cycle (2.84 ± 0.49 and 1.56 ± 1.07, respectively; *P* < 0.05). There was an effect of ewe breed on Neu5Gc, Neu5Ac, Neu5,7Ac_2_, Neu5Gc,9Ac_,_ Neu5,8Ac_2_, and Neu5,9Ac_2_ (*P* < 0.05, Figure 2). At a natural estrus, Suffolk had a lower concentration of Neu5Gc compared with Romanov (*P* < 0.05). Regarding the acetylated species, Suffolk and Ile de France had higher abundance of Neu5Ac compared with Romanov (*P* < 0.001). Belclare had lower concentration of Neu5,7Ac_2_ compared with Fur ewes (*P* < 0.05). Ile de France had lower abundance of Neu5Gc,9Ac than Fur and NWS (*P* < 0.05). However, Ile de France had higher proportion of Neu5,8Ac_2_ compared with Belclare (*P* < 0.05). In addition, Romanov had the lowest concentration of Neu5,9Ac_2_, showing significant differences with Suffolk, Belclare, Ile de France, and Fur. The low fertility Suffolk breed had the highest concentration of Neu5,9Ac_2_, and was higher than both Ile de France (medium fertility) and NWS (high fertility; *P* < 0.05) at both types of cycle.

### Experiment 3: the cervical expression of mucin and sialic acid related genes is affected by ewe breed and type of estrus cycle

Differential gene expression analysis indicated that ewe breed, type of the cycle and their interaction was associated with extensive alterations in cervical gene expression at the follicular phase of the estrus cycle.

#### Mucin genes

**Figure 3:**
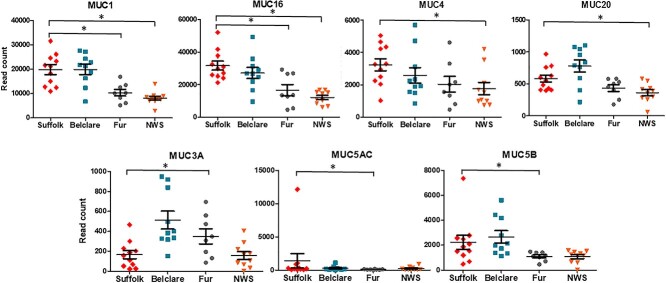
Expression of genes (read counts) at the follicular phase of a synchronized estrus cycle in Suffolk, Belclare, Fur, and Norwegian White Sheep (NWS). Transcript counts were modelled by fitting the data to a negative binomial distribution using genewise dispersion estimates and differentially expressed genes were identified with a generalized linear model likelihood ratio test. Statistical tests were corrected for multiple testing using the Benjamini–Hochberg method. Significant differences (*P* < 0.05) between the reference level (Suffolk) and the other ewe breeds are denoted with a * symbol.

**Figure 4:**
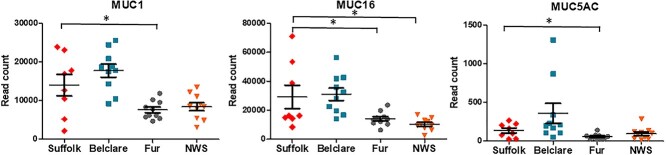
Expression of genes (read counts) at the follicular phase of a natural estrus cycle in Suffolk, Belclare, Fur, and Norwegian White Sheep (NWS). Transcript counts were modelled by fitting the data to a negative binomial distribution using genewise dispersion estimates and differentially expressed genes were identified with a generalized linear model likelihood ratio test. Statistical tests were corrected for multiple testing using the Benjamini–Hochberg method. Significant differences (*P* < 0.05) between the reference level (Suffolk) and the other ewe breeds are denoted with a * symbol.

Transcripts of nine mucin genes encoding secreted and membrane-bound mucins were identified in the sheep cervix. Of these, seven were significantly different between breeds. Mucin 1 (*MUC1*) had higher expression in Suffolk compared with Fur and NWS at the synchronized cycle (*P* < 0.05; Figure 3). At the natural cycle, Suffolk ewes also had expression levels of *MUC1* compared with Fur (*P* < 0.05; Figure 4). There was higher expression of mucin 16 (*MUC16*) in Suffolk compared with Fur and NWS at both a natural and a synchronized cycle (*P* < 0.05). Mucin 4 (*MUC4*) and mucin 20 (*MUC20*) had higher expression in Suffolk compared with NWS at the synchronized cycle only (*P* < 0.05). However, mucin 3A (*MUC3A*) had lower expression in Suffolk compared with Fur at the synchronized cycle (*P* < 0.05). The two gel-forming mucins, *MUC5AC* and *MUC5B* had higher expression in Suffolk compared with Fur at the synchronized cycle (*P* < 0.05). At the natural cycle, Suffolk also had higher expression of the *MUC5AC* compared with Fur ewes (*P* < 0.05).

#### Sialyltransferases

The sialyltransferases are a representative group that catalyze the transfer of sialic acids to the glycan structure. There were 14 sialyltransferases expressed in sheep cervical tissue at the follicular phase of both types of cycle (natural and synchronized), these were *ST3GAL1, ST3GAL2, ST3GAL3, ST3GAL4, ST3GAL5, ST3GAL6, ST6GAL1, ST6GAL2, ST6GALNAC1, ST6GALNAC2, ST6GALNAC3, ST6GALNAC4, ST8SIA2*, and *ST8SIA4*.

**Figure 5:**
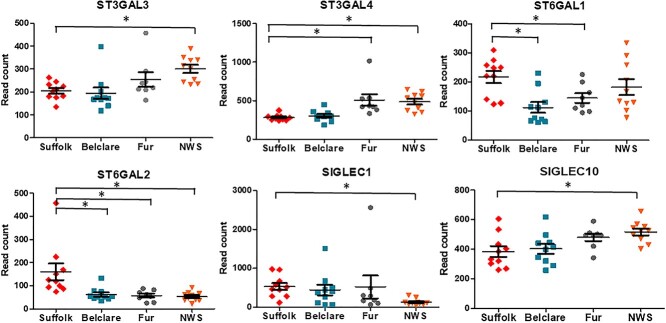
Expression of genes (read counts) at the follicular phase of a synchronized estrus cycle in Suffolk, Belclare, Fur, and Norwegian White Sheep (NWS). Transcript counts were modelled by fitting the data to a negative binomial distribution using genewise dispersion estimates and differentially expressed genes were identified with a generalized linear model likelihood ratio test. Statistical tests were corrected for multiple testing using the Benjamini–Hochberg method. Significant differences (*P* < 0.05) between the reference level (Suffolk) and the other ewe breeds are denoted with a * symbol.

The ST3 *β*-Galactoside *α*-2,3-Sialyltransferase 3 (*ST3GAL3*) that catalyzes the formation of the Neu5Ac-*α*-2,3-Gal-*β*-1,3-GlcNAc, had lower expression in Suffolk compared with NWS (highest fertility) at the synchronized cycle (*P* < 0.05; Figure 5). *ST3GAL4,* which catalyzes the formation of the Neu5Ac-*α*-2,3-Gal-*β*-1,4-GlcNAc and Neu5Ac-*α*-2,3-Gal-*β*-1,3-GlcNAc, had lower expression in the low fertility Suffolk compared with the high fertility Fur and NWS at the synchronized cycle (*P* < 0.05). ST6 *β*-Galactoside *α*-2,6-Sialyltransferase 1 (*ST6GAL1*) transfers sialic acid from the donor of substrate CMP-sialic acid to galactose containing acceptor substrates. There was higher expression in Suffolk compared with Belclare and Fur at the synchronized cycle (*P* < 0.05). In addition, *ST6GAL2* presented higher expression in Suffolk compared with the other three breeds (Belclare, Fur, and NWS) at a synchronized cycle (*P* < 0.05). *ST8SIA2* is involved in polysialic acid synthesis by adding *α*-2,8-linkages to the *α*-2,3-linked and *α*-2,6-linked sialic acid of glycans. There was higher expression of *ST8SIA2* in the low fertility Suffolk breed compared with Fur ewes at both a natural and a synchronized cycle (*P* < 0.05).

#### Sialidases

Sialidases (neuraminidases) are responsible for cleaving the terminal sialic acid of *O*-glycans. Two neuraminidases (*NEU1* and *NEU3*) were identified; however, there was no effect of ewe breeds or type of the cycle in their expression (*P* > 0.05).

#### Sialic acid-specific *O*-acetylesterases

Sialic acid-specific *O*-acetylesterases, including the *SIAE* gene, remove 9-*O*-acetylation modifications from sialic acids and thus permits *α*-2,6 linked sialic acid on *N*-glycans on B cell glycoproteins to interact with CD22/SIGLEC2, a sialic acid-binding lectin that can inhibit B cell antigen receptor signaling. In our study, there were no differences in the expression of *SIAE* between ewe breeds at either a natural or synchronized cycle (*P* > 0.05). However, *CD22* had over twofold increased expression (FC = 2.71) in Suffolk compared with NWS at the synchronized cycle (*P* < 0.05).

#### Sialic acid-binding immunoglobulin-like lectins (Siglecs)

**Figure 6:**
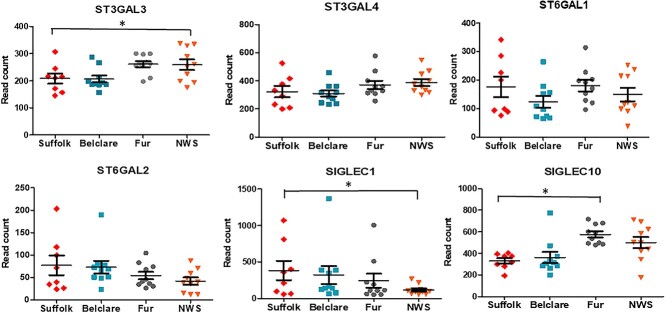
Expression of genes (read counts) at the follicular phase of a natural estrus cycle in Suffolk, Belclare, Fur, and Norwegian White Sheep (NWS). Transcript counts were modelled by fitting the data to a negative binomial distribution using genewise dispersion estimates and differentially expressed genes were identified with a generalized linear model likelihood ratio test. Statistical tests were corrected for multiple testing using the Benjamini–Hochberg method. Significant differences (*P* < 0.05) between the reference level (Suffolk) and the other ewe breeds are denoted with a * symbol.

We identified four types of Siglecs (*SIGLEC1*, *SIGLEC10*, *SIGLEC14*, and *SIGLEC8*) at the synchronized estrus cycle although at the natural cycle we did not detect the expression of *SIGLEC8*. *SIGLEC1* had over twofold increased expression in Suffolk compared with NWS at both the natural and synchronized cycle (*P* < 0.05). However, *SIGLEC10* had decreased expression in Suffolk compared with Fur at the natural cycle (*P* < 0.05; Figure 6).

### Experiment 4: the percentage of goblet cells differs between high and low fertility ewe breeds under the effect of estrus synchronization

**Figure 7:**
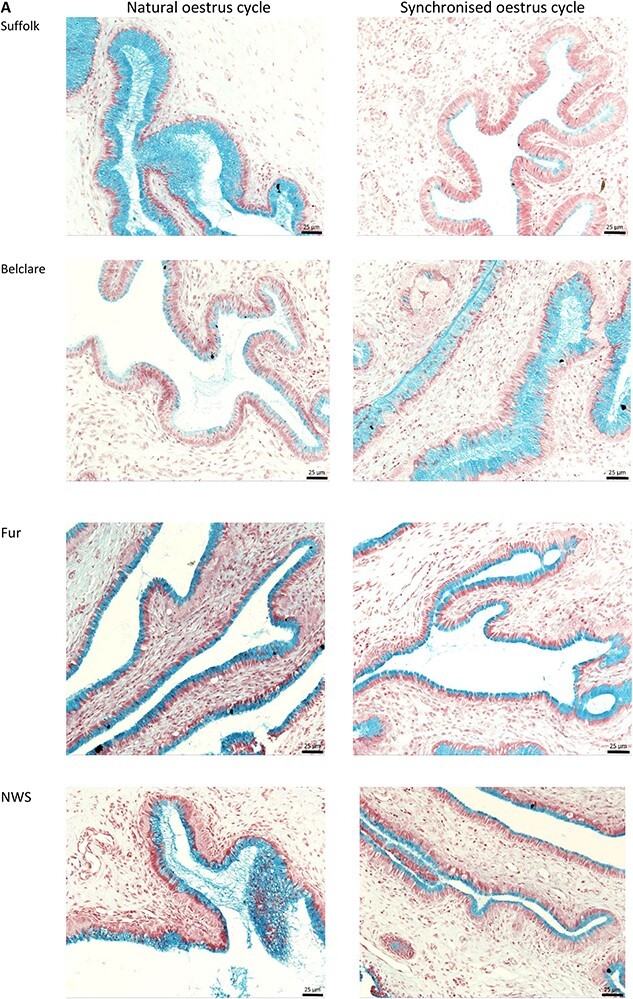

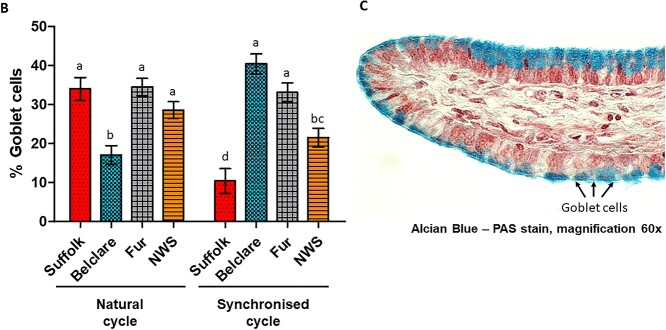
(A) Representative image of a cervical tissue section from each of the experimental groups (Suffolk, Belclare, Fur, and Norwegian White Sheep (NWS)) at both a natural and a synchronized estrus cycle. Goblet cells were visualized using Alcian blue pH 2.5 (AB pH 2.5) and periodic acid Schiff (PAS) stain. (B) Percentage (%) of goblet cells in a defined epithelial area of cervical tissue from Suffolk, Belclare, Fur, and NWS ewes at the follicular phase of a natural and a synchronized estrus cycle. Multivariate analysis of variance (MANOVA) with post-hoc analysis using Tukey’s Honest Significant difference test was performed to identify significant differences across groups. Values are mean ± SEM. ^abc^ Different superscripts differ significantly (*P* < 0.05). (C) Image of a cervical tissue section (60× magnification).

There was a type of the cycle (natural versus synchronized) by ewe breed interaction on the percentage of goblet cells (*P* < 0.05; Figure 7). During the natural cycle, Belclare ewes had the lowest percentage of goblet cells and it was lower than in Suffolk, Fur, and NWS (*P* < 0.05). However, in the synchronized cycle, Suffolk ewes presented the lowest percentage of goblet cells, which was lower compared with Belclare, Fur, and NWS ewes (*P* < 0.05). There were no differences between both Norwegian ewe breeds (Fur and NWS) with the natural cycle whereas, during the synchronized cycle Fur ewes had a higher percentage of goblet cells in the cervical epithelium than NWS (*P* < 0.05). The effect of estrus synchronization was also evident as Suffolk had a lower percentage of goblet cells than Fur and NWS at the synchronized cycle (*P* < 0.05) although there were no differences between them at the natural cycle (*P* > 0.05).

## Discussion

This study has characterized, for the first time, the sialylated cervical mucins from ewe breeds with previously known differences in pregnancy rates following cervical AI with frozen–thawed semen [13, 14]. We used a combination of biochemical and molecular techniques such as RP-UPLC, WAX-UPLC, and RNA-sequencing to reveal differences in sialic acid composition and the expression of key genes involved in mucin and sialic acid biosynthesis between ewe breeds at the follicular phase of both natural and synchronized estrus cycles. We identified higher levels of Neu5,9Ac_2_ in the low fertility Suffolk breed compared with Ile de France (medium fertility) and NWS (high fertility). In addition, the percentage of cervical goblet epithelial cells differed between high and low fertility ewe breeds under the effect of exogenous hormones for estrus synchronization since Suffolk ewes had lower percentage of goblet cells than Fur and NWS. Gene expression analysis identified higher expression of *MUC5AC*, *MUC5B*, *ST6GAL1*, and *ST6GAL2* and lower expression of *ST3GAL3, ST3GAL4*, and *SIGLEC10* in Suffolk compared with high fertility ewe breeds. In summary, this study has shown ewe breed differences in the sialic acid composition and cervical gene expression, which are likely to at least partly explain why cervical AI with frozen–thawed semen works in Norway but not elsewhere. Unacceptably low pregnancy rates following cervical AI using frozen–thawed semen could be attributed to higher sialic acid content leading to a greater adherence of sperm to large mucins within the cervix.

We identified nine mucin genes encoding two types of mucins: gel-forming mucins (*MUC5AC* and *MUC5B*) and membrane-bound mucins (*MUC1, MUC13, MUC16, MUC17, MUC20, MUC3A*, and *MUC4*). In the present study, we identified higher expression of *MUC1* in the low fertility Suffolk compared with Fur at both a natural and a synchronized cycle. It is known that *MUC1* plays a role as an antimicrobial barrier [26]. Thus, the low fertility Suffolk breed could have a higher cervical bacteria load, which may be one of the reason of the poor pregnancy rates.

In the cervix, the expression of gel-forming mucins increase around the time of ovulation across a range of species, including the cow [3], sheep [17], and human [5]. We identified that the low fertility Suffolk breed had higher expression of *MUC5AC* compared with Fur ewes (high fertility) at both types of cycle. This is in agreement with a previous study in sheep by Richardson et al. [17], where low fertility Suffolk ewes had higher expression of *MUC5AC* than medium fertility Belclare ewes. The role of *MUC5AC* in sperm transport is unknown, however, it seems to be correlated with mucus rheology since in our previous study on the same animals [18], Suffolk ewes had less viscous mucus than Fur ewes at the follicular phase of both a synchronized and a natural cycle, although mucus viscosity values were also low in NWS (highest fertility). These results support the hypothesis of a negative correlation between mucin content and mucus viscosity although this does not appear to be correlated with the pregnancy rates previously reported following cervical AI with frozen–thawed semen.

We also assessed the percentage of goblet cells, which was lower in Suffolk ewes compared with the other ewe breeds at the synchronized cycle although the expression of gel-forming mucins was up-regulated in Suffolk as we reported above. This suggests that the higher expression of gel-forming mucins in the cervix of Suffolk ewes (low fertility) was not complemented with higher percentage of goblet cells, thus pointing to an imbalance of mucin synthesis and secretion in this breed. Estrus synchronization has been shown to increase the production as well as alter the biochemical and proteomic composition of ovine cervical mucus in the lead up to ovulation [27, 28]. We previously reported an increased rate of mucus production following synchronization in the animals used in this study [18]. Although there was no ewe breed by estrus type interaction in mucus production or gross mucus viscosity there could be more subtle differences in the sialic acid composition that are affected by ewe breed and or synchronization.

Cervical mucus around the time of ovulation has been previously characterized by the presence of nonsialylated glycans as the most prevalent structures in cervical mucus from women [5]. Our results from the WAX-UPLC analysis showed a trend that high fertility ewe breeds have higher levels of nonsialylated glycans in cervical mucus irrespective of whether their cycle was synchronized or natural. Although more biological replicates would be needed to conclude this, it appears that low sialic acid content in cervical mucus may promote sperm transport as was recently demonstrated by Richardson et al. [17].

We also investigated the expression of the sialyltransferases. For example, the low fertility Suffolk had lower expression of *ST3GAL3* and *ST3GAL4* compared with NWS (high fertility) at the synchronized cycle. In addition, expression of *ST3GAL4* was lower in Suffolk compared with Fur ewes (high fertility) at the synchronized cycle. However, we observed higher expression of *ST6GAL2* in Suffolk compared with Belclare, Fur, and NWS at the synchronized cycle. In addition, there was higher expression of *ST6GAL1* in Suffolk compared with Belclare and Fur ewes at the synchronized cycle. Given the aforementioned ewe breed effects on the sialylatransferases gene expression and the fact that AI is performed to a natural estrus in Norway, but to a synchronized estrus in other countries, we hypothesized that estrus synchronization could be one of the reasons for impaired cervical sperm transport. Richardson et al. [17] also observed higher expression of *ST6GAL1* in the low fertility Suffolk breed at the synchronized cycle, which was correlated with higher abundance of sialic acid and lower number of sperm in the cervical channels of Suffolk compared with Belclare ewes. These results suggest that Neu5Ac/Neu5G-*α*-2,6 sialic acid could be involved in the inhibition of sperm migration through the cervical mucus, which is more evident at the synchronized cycle.

In addition, Richardson et al. [17] showed that the addition of free sialic acid (6′-sialyllactose (Neu5Ac-*α*-2,6-Gal-*β*-1,4-Glc)) increased the ability of sperm to penetrate the ovine cervical mucus *in vitro*. This demonstrates that sperm become attached to the sialic acid of the large mucin glycoproteins, which may be mediated via a sialic-acid-recognizing protein on the sperm surface [29, 30]. Thus the addition of free sialic acid blocked the sialic-acid-recognizing receptors on the sperm surface thereby preventing sperm from binding to the sialic acid of the cervical mucins. Therefore, the *α*-2,6 linked sialic acid on cervical mucins appear to be involved in the attachment of sperm to cervical mucins thus inhibiting the progression of sperm through the cervix. There are only a few studies on the alteration of the sperm glycocalyx during cryopreservation. Wu et al. [31] demonstrated that the content of sialic acid on the surface of frozen–thawed human sperm was significantly decreased.

The linkage of sialic acid terminal to the glycan chain seems to be important for sperm transport as we outlined above, however we also studied if the sialic acid species differ significantly between high and low fertility ewe breeds. From over 50 different sialic acid structures in nature, we characterized seven. The two most prevalent are *N*-acetyl-neuraminic acid (Neu5Ac) and *N*-glycolyl-neuraminic acid (Neu5Gc), acetylated and glycosylated, respectively [8]. This is consistent with the current study where both moieties were also identified as the major species of sialic acid in sheep cervical mucus. Levels of Neu5,9Ac_2_ (9-*O*-acetyl-*N*-acetylneuraminic acid) were highest in the low fertility Suffolk breed and significantly higher than in Ile de France (medium fertility) and NWS (high fertility). Robinson et al. [32] demonstrated that with the presence of 9-*O*-acetylated sialic acid, the mucus layer become more susceptible to the action of bacterial sialidases. Therefore, this suggests that the low fertility Suffolk breed could be more exposed to mucus degradation and thus bacterial infection due to the high levels of Neu5,9Ac_2_, which could result in poor pregnancy rates.

It is well known that there are sialic acid receptors on the sperm surface, which mediate the interaction of sperm with the cervix and its secretions. We identified for the first time in the sheep cervix the expression of four Siglec genes (*SIGLEC1, SIGLEC10, SIGLEC14*, and *SIGLEC8*). Of these genes, *SIGLEC10* showed lower expression in the low fertility Suffolk breed compared with Fur ewes (high fertility) at the natural cycle. *SIGLEC10* is expressed on B cells, NK cells and circulating monocytes in human [33]. SIGLEC10 and its ligand CD24 are required to inhibit the production of pro-inflammatory cytokines [34]. In addition, it has described that SIGLEC10-CD24 interaction selectively represses the NFk-*B* driven inflammatory response to danger-associated molecular patterns [34]. Therefore, the lower expression of *SIGLEC10* in the low fertility Suffolk breed suggests an inflammatory environment in the cervix of the Suffolk that could result in reduced cervical sperm transport. On the other hand, *SIGLEC1* showed an up-regulation in Suffolk compared with NWS (high fertility) at both types of estrus cycle. *SIGLEC1* has been reported to be up-regulated upon viral infection through the IFN/JAK/STAT1 signaling pathway [35], which spreads the infection and helps virus to escape from neutralization. Thus, *SIGLEC1* upregulation in the Suffolk seems to be involved in evading host immune response by different families of viruses.

In conclusion, this study suggests that *α*-2,6 linked sialic acid and Neu5,9Ac_2_ on cervical mucins may be involved in the interaction with Siglecs on the sperm surface, thus causing sperm to become immobilized in the cervical mucins. Further *in vitro* and *in vivo* functional studies are required to understand the precise role of specific sialic acid terminals and Siglecs in cervical sperm transport.

## Supplementary Material

L_Abril-Parreno_et_al_sialylated_cervical_mucins_sup_fig_ioac077

L_Abril-Parreno_et_al_sialylated_cervical_mucins_table1_ioac077

L_Abril-Parreno_et_al_sialylated_cervical_mucins_Sup_file_1_ioac077

L_Abril-Parreno_et_al_sialylated_cervical_mucins_sup_file2_ioac077

## Data Availability

The datasets generated and/or analyzed during the current study are available in the NCBI Gene Expression Omnibus https://www.ncbi.nlm.nih.gov/geo/ under accession number GSE179486.
