# Supplementary material for: Biochemical and molecular characterization of sialylated cervical mucins in sheep
Source: Biol Reprod. 2022 Apr 26;107(2):419–31. doi: 10.1093/biolre/ioac077 (PMC9382375; doi:10.1093/biolre/ioac077)
Supplement: L_Abril-Parreno_et_al_sialylated_cervical_mucins_sup_fig_ioac077 [file l_abril-parreno_et_al_sialylated_cervical_mucins_sup_fig_ioac077.docx]

# SUPPLEMENTAL FIGURE

# Title: Biochemical and molecular characterisation of sialylated cervical mucins in sheep

**Authors:** Laura Abril-Parreño, Jack Morgan, Anette Krogenæs, Xavier Druart, Paul Cormican, Mary E Gallagher, Colm Reid, Kieran Meade, Radka Saldova and Sean Fair


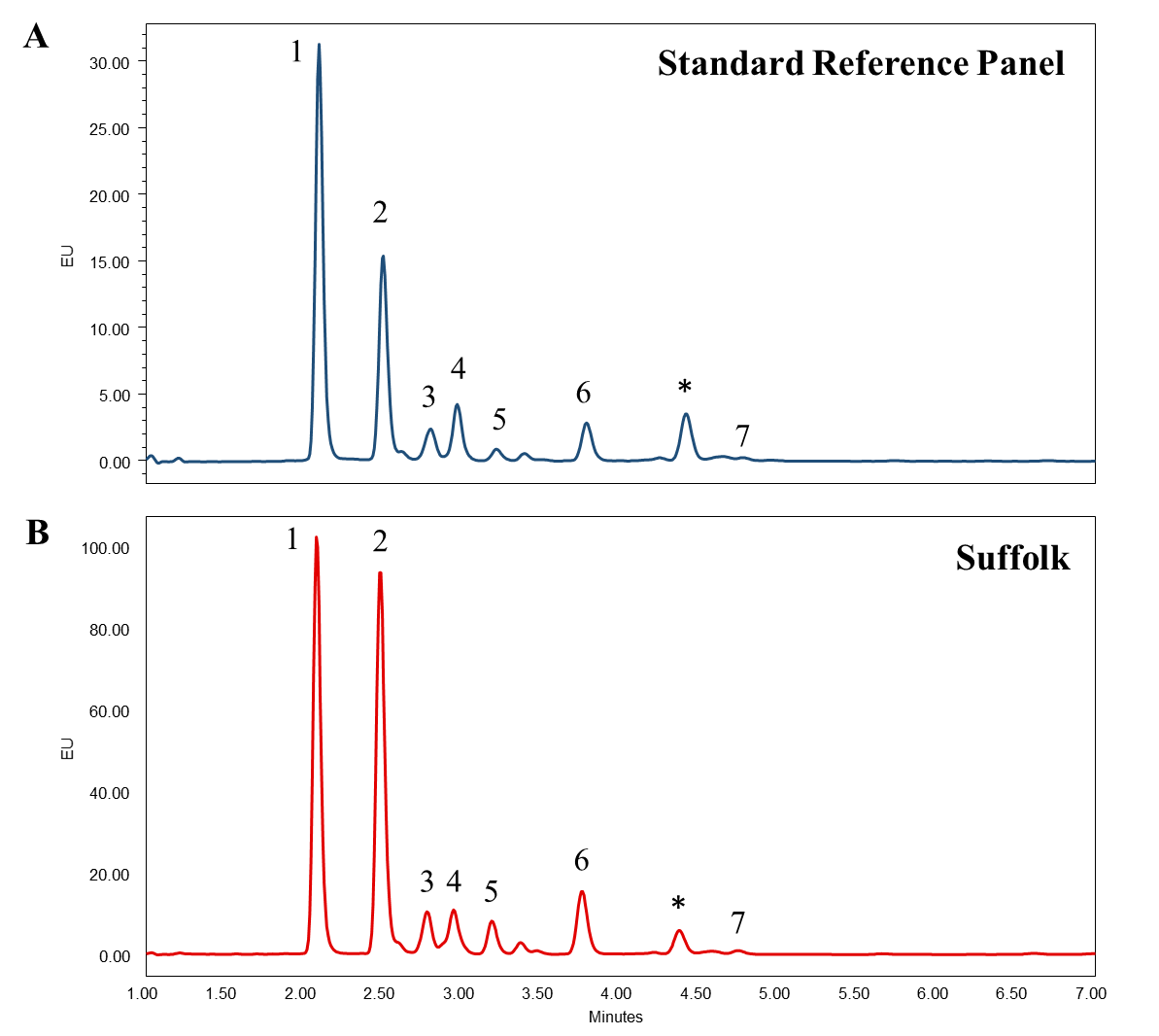


Supplemental Figure S1. (A) The 1,2-diamino-4,5-methylenedioxybenzene (DMB) labelled sialic acid reference panel analysed by reverse phase (RP) - Ultra-performance liquid chromatography (UPLC). (B) RP-UPLC chromatogram from the Suffolk ewe breed at the follicular phase of a synchronised oestrous cycle run on the LudgerSep-uR2 UPLC column. Peaks: 1 = Neu5Gc; 2 = Neu5Ac; 3 = Neu5,7Ac_2_; 4 = Neu5Gc,9Ac_2_; 5 = Neu5,8Ac_2_; 6 = Neu5,9Ac_2_; 7= Neu5x,xAc3 (where x is an unknown acetyl position); * = Reagent.
