# Supplementary material for: Biochemical and molecular characterization of sialylated cervical mucins in sheep
Source: Biol Reprod. 2022 Apr 26;107(2):419–31. doi: 10.1093/biolre/ioac077 (PMC9382375; doi:10.1093/biolre/ioac077)
Supplement: L_Abril-Parreno_et_al_sialylated_cervical_mucins_table1_ioac077 [file l_abril-parreno_et_al_sialylated_cervical_mucins_table1_ioac077.docx]

Table 1. Relative percentage area (%) derived from the UPLC total profile of nonsialylated (Fraction S0), mono-sialylated (Fraction S1), di-sialylated (Fraction S2) and tri-sialylated (Fraction S3) *O*-glycans in cervical mucin from six ewe breeds (Suffolk, Belclare, Ile de France, Romanov, Fur and Norwegian White Sheep (NWS) at the follicular phase of both a natural and a synchronised oestrus cycle. One pooled samples per breed and type of cycle was analysed by Weak Anion exchange chromatography (WAX) – Ultra-Performance Liquid Chromatography (UPLC) analysis.

| **Ewe Breed** | **Type of cycle** | **Fraction S0**  **(%)** | **Fraction S1**  **(%)** | **Fraction S2**  **(%)** | **Fraction S3**  **(%)** |
| --- | --- | --- | --- | --- | --- |
| Suffolk | Natural | 69.9 | 23.9 | 5.1 | 1.2 |
| Suffolk | Synchronised | 79.6 | 17.0 | 2.6 | 0.7 |
| Belclare | Natural | 76.6 | 18.9 | 3.4 | 1.1 |
| Ile de France | Natural | 71.5 | 23.2 | 4.3 | 1.0 |
| Ile de France | Synchronised | 71.5 | 22.9 | 4.6 | 1.0 |
| Romanov | Natural | 64.7 | 28.1 | 6.2 | 0.9 |
| Romanov | Synchronised | 79.4 | 17.5 | 2.5 | 0.6 |
| Fur | Natural | 86.5 | 11.2 | 1.8 | 0.4 |
| Fur | Synchronised | 72.2 | 23.1 | 3.9 | 0.7 |
| NWS | Natural | 82.6 | 13.2 | 2.3 | 1.8 |
| NWS | Synchronised | 91.4 | 7.0 | 1.2 | 0.4 |

*Belclare breed at the synchronised cycle was not analysed as there was insufficient amount of mucin remaining to perform this analysis. NWS = Norwegian White Sheep.
