# Supplementary material for: Biochemical and molecular characterization of sialylated cervical mucins in sheep
Source: Biol Reprod. 2022 Apr 26;107(2):419–31. doi: 10.1093/biolre/ioac077 (PMC9382375; doi:10.1093/biolre/ioac077)
Supplement: L_Abril-Parreno_et_al_sialylated_cervical_mucins_sup_file2_ioac077 [file l_abril-parreno_et_al_sialylated_cervical_mucins_sup_file2_ioac077.docx]

**SUPPLEMENTAL FILE 2**

# Title: Biochemical and molecular characterisation of sialylated cervical mucins in sheep

**Authors:** Laura Abril-Parreño, Jack Morgan, Anette Krogenæs, Xavier Druart, Paul Cormican, Mary E Gallagher, Colm Reid, Kieran Meade, Radka Saldova and Sean Fair

**Mucin purification protocol (Experiment 1 and 2) previously described by Abril-Parreño, et al. [1]**

Mucus samples were ranked according to viscosity at collection (as assessed by the time to fill a 20 µm deep chamber slide) and following thawing were pooled according to viscosity post collection to ensure a sufficient yield of mucins for analysis. Each pool consisted of mucus from five ewes (five most viscous pooled together, then the next five and so on) of the same breed collected over three replicates (total of 15 samples pooled across the five ewes). The volume of mucus in each pool/group was ~ 5 mL. Each pooled sample was mixed with an equal volume of 8 M guanidine hydrochloride (GdnCl; Sigma Aldrich, Arklow, Co Wicklow, Ireland) in order to solubilize the mucus and homogenise the samples (the final volume was made up to 30 mL). Dithiothreitol (DTT) (Sigma Aldrich) was added to a final concentration of 10 mM and incubated at 37 °C for 5 h. Iodacetamide (Sigma Aldrich) was added to a final concentration of 25 mM and the samples were incubated at room temperature overnight. The isopycnic density gradient centrifugation was carried out in CsCl/GdnCl. The density of the samples was adjusted to 1.40 g/mL with CsCl in Beckman Ultra-clear tubes which were centrifuged at 65,000 rpm for 18 h at 10 °C in an Optima L-100 XP (Beckman Coulter Inc, Brea, USA) ultracentrifuge using the 70 Ti rotor without break. The density gradient created was unpacked in 1 mL fractions from the top to the bottom of the tube. Following this duplicate 5 μL aliquots from each fraction were blotted onto a polyvinylidene difluoride membrane using a Whatmann manifold Slot blot apparatus (Schleicher & Schuell, Inc, Keene, NH) and stained with periodic acid –Schiff stain (PAS staining (VWR, Radnor, Pennsylvania, USA) in order to assess the relative intensity of carbohydrate in the sample. The carbohydrate rich fractions observed from the slot blot which also had a density profile between 1.35 to 1.45 g/mL were pooled and then separated by size exclusion chromatography. The samples were loaded on a Sepharose CL-4B column (Sigma Aldrich) and eluted with 50 mM Tris/100 mM KCl, pH 7.5, as mobile phase. The eluate was collected in fractions of 4 mL using a fraction collector and then a sample (~20 µL) of all fractions was slot blotted and stained with PAS as described before. Mucin rich fractions were pooled and freeze-dried, then resuspended in H2O and desalted on a Bio-gel P6 column (Bio-Rad Laboratories, Hercules, CA). Fractions collected with the fraction collector were analysed by slot blotting and PAS staining (40 µL), then carbohydrate rich fractions were pooled and freeze-dried. The freeze dried material was used as the final pool of purified mucins from each sample and was weighed and stored at -20 °C until analysis.

**Reference**

1. Abril-Parreño L, Wilkinson H, Krogenæs A, Morgan J, Gallagher ME, Reid C, Druart X, Fair S, Saldova R. Identification and characterization of O-linked glycans in cervical mucus as biomarkers of sperm transport: A novel sheep model. Glycobiology 2021.
